# Pharmacokinetic profiles of artesunate following multiple intravenous doses of 2, 4, and 8 mg/kg in healthy volunteers: Phase 1b study

**DOI:** 10.1186/1475-2875-11-255

**Published:** 2012-08-01

**Authors:** Robert Scott Miller, Qigui Li, Louis R Cantilena, Kevin J Leary, George A Saviolakis, Victor Melendez, Bryan Smith, Peter J Weina

**Affiliations:** 1 Department of Drug Discovery, Division of Experimental Therapeutics, Walter Reed Army Institute of Research, 503 Robert Grant Avenue, Silver Spring, MD 20910 USA; 2 Uniformed Services University of the Health Sciences (USUHS), Bethesda, MD, USA; 3 US Army Medical Material Development Activity, Ft Detrick, MD, USA

**Keywords:** Artesunate injection, Severe malaria, Pharmacokinetics, Healthy volunteers, Tolerability, Multiple doses

## Abstract

**Background:**

Severe malaria results in over a million deaths every year, most of them in children aged less than five years and living in sub-Saharan Africa. Injectable artesunate (AS) was recommended as initial treatment for severe malaria by WHO in 2006. The Walter Reed Army Institute of Research (WRAIR) has been developing a novel good manufacturing practice (GMP) injection of AS, which was approved by the US FDA for investigational drug use and distribution by the CDC.

**Methods:**

Tolerability and pharmacokinetics of current GMP intravenous AS, as an anti-malarial agent, were evaluated after ascending multiple doses of 2, 4, and 8 mg/kg daily for three days with 2-minute infusion in 24 healthy subjects (divided into three groups) in the Phase 1 clinical trial study.

**Results:**

Results showed that there were no dose-dependent increases in any adverse events. Drug concentrations showed no accumulation and no decline of the drug during the three days of treatment. After intravenous injection, parent drug rapidly declined and was converted to dihydroartemisinin (DHA) with overall mean elimination half-lives ranging 0.15-0.23 hr for AS and 1.23-1.63 hr for DHA, but the peak concentration (C_max_) of AS was much higher than that of DHA with a range of 3.08-3.78-folds. In addition, the AUC and C_max_ values of AS and DHA were increased proportionally to the AS climbing multiple doses.

**Discussion:**

The safety of injectable AS, even at the highest dose of 8 mg/kg increases the probability of therapeutic success of the drug even in patients with large variability of parasitaemia.

## Background

Artemisinin and its derivative act rapidly against drug-resistant *Plasmodium falciparum* strains, and are widely used for the treatment of various malarias in humans. Dihydroartemisinin (DHA) is originally obtained by sodium borohydride reduction of artemisinin, an endoperoxide containing sesquiterpene lactone, which was isolated by Chinese researchers and characterized as the antimalarial principle of the plant *Artemisia annua*. *In vitro* bioassay tests have shown DHA to be more potent than artemisinin. DHA is similar to artesunate (AS) [1] and three to five-fold more active and more toxic than other artemisinin derivatives [2,3]. It can completely inhibit parasite growth within two to four hours, and is the only artemisinin derivative with activity against all asexual blood stage parasites [4]. The excellent effectiveness of AS by rapid parasite and fever clearance has been mostly attributed to its rapid and extensive hydrolysis to DHA [5-8].

Artemisinins have been used in malaria treatment as monotherapy since 1983. However, monotherapy with artemisinin derivatives was significantly discouraged after 2001 to prevent the emergence of resistance, and the new policy is to treat non-severe malaria with artemisinin derivatives in combination with other anti-malarials [9]. For complicated and severe malaria however, intravenous AS, as initial monotherapy, is still a first-line treatment for both adults and children in Asian countries [9] and some areas in Africa [10]. Severe malaria is generally defined as acute malaria with major signs of organ dysfunction or high levels of parasitemia. Severe malaria, if not treated, results in 100% mortality [11]. Practically though, severe malaria can be defined as acute malaria where the patient cannot take oral medications to effect a cure. In areas where malaria is endemic, young children and pregnant women are at high risk of severe malaria. It is fundamental that plasma concentrations of a highly effective anti-malarial drug are achieved as rapidly as possible.

After recent clinical studies with severe malaria patients, intravenous AS was determined to be the drug with the highest treatment success and the lowest incidence of adverse events [9,12,13]. The two largest trials ever conducted for severe malaria in endemic regions showed that, in both adults and children, treatment with intravenous AS is superior to intravenous quinine. The mortality rate among quinine-treated patients was 22% and 10.9% in the SEAQUAMAT and AQUAMAT studies respectively; for AS this was 15% and 8.5%, a significant reduction of 35% and 22.5% respectively [13,14]. Patients with hyperparasitaemia (>10% of RBC) had a significantly greater treatment effect with AS than non-hyperparasitaemia patients. Intravenous AS was also better tolerated, safer and easier to use than quinine. The life-saving benefit of AS in severe malaria was recognized by WHO in 2006 and since then intravenous AS has been the treatment of choice for severe falciparum malaria [12,13].

Many other clinical studies for pharmacokinetic evaluation of AS have been conducted, mostly in Asia and Africa [15]. Peak concentration (C_max_) has been demonstrated to be more important than AUC in producing the improved efficacy of anti-malarial drugs as outlined in previous pharmacokinetics (PK) and pharmacodynamics (PD) evaluation [16]. The AS, which is the only artemisinin derivative clinically available as an injectable formulation, can provide sufficiently high peak concentrations in the patients and can provide the most rapid efficacy in parasite killing, showing that injectable AS is pharmacokinetically and pharmacodynamically superior when compared to other artemisinins [16]. Therefore, AS monotherapy is still needed, if only for this single niche indication [17,18].

The Chinese manufacturer (Guilin Pharmaceutical Company Ltd, Shanghai, China) of the product that was used in most of the clinical trial studies and treatment cases recently improved the production process, with support of the Medicines for Malaria Venture (MMV). However, only intravenous quinine or quinidine is still used in Europe and the USA because the main barrier for the use of intravenous AS is the absence of a product that is manufactured under good manufacturing practices (GMP). Since 2004, the Walter Reed Army Institute of Research (WRAIR) has been developing a novel GMP injection of AS, which was approved by the US FDA for investigational drug use and distribution by the CDC [19]. As previously reported, a Phase 1a clinical trial of this GMP product was conducted, with single dose (0.5, 1, 2, 4, and 8 mg/kg) administration of intravenous artesunate studied in 40 healthy volunteers [20]. The present Phase 1b study (ClinicalTrials.gov Identifier: NCT00292942) describes the PK of injectable AS, using escalating multiple doses in healthy volunteers daily for three days with the aim of developing a safe and feasible dose.

## Methods

### Chemicals

The bulk artesunic acid [4-(10’dihydro-artemisinin-oxymethyl) succinate] substance was purchased from Knoll AG (Switzerland). BASF Pharmaceuticals rebottled it from the original company (GMP). The clinical trial AS (Batch #: 14462–16) has been tested for sterility and short-term stability. The formulation is contained in sterilized bottles with 110 mg artesunic acid per bottle. The injection buffer for AS is manufactured as a GMP phosphate salt with 0.3 M PBS (pH 8.0) and provided by Stanford Research Institute (Menlo Park, CA, USA). The multiple dosage form for the Phase 1 clinical trial was manufactured to be prepared as follows: 110 mg by reconstitution in 11 ml of this buffer (10 mg/ml) to make a fresh stock solution sufficient to dose all the volunteers scheduled daily on three given days.

### Subject background

For confirmation of their health status, all subjects were selected to be healthy adult males (seventeen) and non-pregnant, non-lactating females (seven) and were assessed by inspecting their full clinical history and conducting appropriate examinations, including clinical laboratory evaluations (clinical chemistry, haematology and urinalysis), thyroid stimulating hormone, free T4, electrocardiograms, and blood pressure before entry into the study. Subjects were also checked for their body mass index (BMI) which was ideally between 18 and 29 kg/m^2^ and, if out of this range, their weight needed to be "not clinically significant" or within 15% of their ideal body weight. All subjects gave written informed consent.

### Study design

The study was a double blind, ascending multiple doses, alternating group, safety, tolerance, and PK study. Injectable AS daily for three days was administered intravenously to three groups of the subjects using alternating doses of 2, 4, and 8 mg. All eight subjects in each dose group received either the AS (six subjects) or placebo (two subjects) in a pre-randomized manner. The study physician, after verifying the code with a study nurse, would then inject either the drug or placebo into an injection port at the pre-measured dose over about 2 min as a short-term intravenous infusion; the study nurse recorded the time of study drug reconstitution, infusion start and completion of injection. When the injection was completed, the study nurse recorded tolerance of injection to include both inspection of site and subjects’ responses. Subjects were provided a light lunch at approximately 4 hours after dosing. Normal activities, excluding strenuous exercise, were permitted four hours after dosing. Blood samples for the measurements of AS and DHA levels in plasma was approximately 10 min before dosing (0 h), and at 5, 20, 40 min, 1, 2, 4, 6, and 8 h on days 1, 2, and 3 post-dose. During the multiple-dose period, samples were collected immediately before morning dose on days 2 and 3 for additional trough values. All the samples were stored at −80 °C until shipment and were then shipped to a bio-analytical GLP laboratory, Midwest Research Institute (MRI), in Kansas City, MO, USA. The human use protocol numbers for the Phase 1 trial are: USUHS #G183RW; WRIAR #1128; and HSRRB #A-13276.

### LC-MS/MS assay

An LC-MS/MS method for the quantitation of AS and DHA (50–100 μl) in human plasma was validated from 2 to 400 ng/ml for AS and DHA. The analytes were extracted from human plasma with ethyl acetate. These extracts were dried and reconstituted in 50:50 (v/v) acetonitrile: water containing indomethacin as the internal standard. The reconstituted extracts were analysed on a Micromass Quattro II Mass Spectrometer in the positive ion electrospray ionization (+ESI) mode. The compounds of AS, DHA, and internal standard, indomethacin, were monitored in the multiple reaction monitoring (MRM) mode. This method employed a Varian Pursuit C_18_ column (150 x 2.0 mm, 5-μm particle size) and a gradient elution with the following mobile phases: A: 10 mM ammonium acetate in water with 1% formic acid and B: 10 mM ammonium acetate in acetonitrile with 1% formic acid, for the chromatographic separation.

Standard curve and quality control (QC) samples were generated by spiking interference-free human plasma samples with known amounts of AS, DHA, and internal standard. The drug concentrations of the QC samples chosen were within the range of the standard curve, and included a lower limit of quantification (LLOQ), low (<3 x LLOQ), medium, and high QC levels. The limit of quantification was 3.4-4.3 ng/ml for AS and 1.7-2.6 ng/ml for DHA. The peak area ratios (PARs) of AS (product at m/z 163.13, from parent ion at m/z 402.10, collision energy 26 V) and DHA (product at m/z 163.13, from parent ion at m/z 302.10, collision energy 20 V) to indomethacin internal standard (product at m/z 139.00, from parent ion at m/z 358.00, collision energy 37 V) were calculated for each sample from the measured peak areas obtained by LC-SRM. Drug concentrations in QC samples and experimental rat plasma samples were calculated by this best-fit equation and the PARs obtained from the LC-MS/MS analysis. Calibration standards and QC samples were analysed to evaluate the performance of the assay.

### Compartmental analysis (CA) of artesunate

Compartmental (model-dependent) and non-compartment (model-independent) PK analysis are common methods to determine PK parameters, which are acceptable by FDA [21]. In order to compare only the clinically relevant concentrations, the compartmental PK analysis was selected for PK analysis in this study and based on a short-term (2 min) intravenous infusion in accordance with a previous PK evaluation [20]. Injectable AS disposition was best described by a two-compartment model with a rapid initial distribution phase after intravenous administration. The input of the drug was assumed to follow zero-order kinetics, and elimination from the central compartment occurred with first-order kinetics. Compartmental analysis of concentration-time data for parent drug was performed using the WinNonlin software with an intravenous infusion program. The first-order method with logarithmic transformation of all drug-concentration data was used throughout.

The drug plasma concentrations at the end of an intravenous (IV) infusion (C_max_) were calculated from the corresponding model equations (IV-infusion model, WinNonlin) at the respective time points. The AUC_(0-t)_ (where t = the time point for the last sample on the PK profile in which quantifiable drug was detected) was estimated using linear or linear/log trapezoidal calculations. The C_max_ and the time to maximum drug concentration (t_max_) were obtained from the experimental values and the predicted AS concentration at the end of the infusion (2 min) was calculated by compartmental modelling. The values of the following PK parameters were derived by a compartmental method for AS with WinNonlin Version 5.2: half-life (t½); mean residence time (MRT); the AUC from time zero to the last sampling time (AUC_t_); the AUC from time zero extrapolated to infinity (AUC_∞_); total systemic clearance (CL); and volume of distribution during steady state (*V*_ss_), which was obtained by adding the volumes of the different compartments (e g, *V*_ss_ = *V*_1_ + *V*_2_). A drug accumulation index was estimated from the AUC values on day 3 (last multiple dose administration) divided by the AUC on day 1 (first multiple dose administration).

### Non-compartmental analysis (NCA) of dihydroartemisinin

Previous PK analysis confirmed that the PK parameters of DHA showed no significant differences in C_max_, AUC, and total clearance over bioavailability (CL/F) between the NCA and CA analyses, indicating that the PK modelling for DHA is model-independent [20]. Therefore, in the present report the non-compartmental method is suitable for DHA. The maximum concentration in plasma for each subject was read directly from the experimental values. For the determination of initial approach to PK parameters of DHA in plasma after systemic application, a non-compartmental analysis was performed using WinNonlin (version 5.2; Pharsight Corp, Mountain View, CA, USA). The values of the following PK parameters were derived by a non-compartmental method: the half-life (t½); mean residence time (MRT); the AUC from time zero to the last sampling time (AUC_t_); the AUC from time zero extrapolated to infinity (AUC_inf_); relative total systemic clearance (CL/F_obs); and volume of distribution during steady state (*V*_ss_/F_obs). A drug accumulation index was estimated from the AUC value on day 3 divided by the AUC value on day 1 (first multiple dose administration). The AUC_inf_ ratios of the metabolite to the parent compound AS were calculated to determine exposure to any metabolite compared with the parent drug.

### Other data evaluation

Statistical analysis was conducted with Microsoft Excel using a Student's t-test for dependent samples to compare means of paired samples between groups. A drug comparison of day 3 AUC value vs. day 1 AUC for each dosing group was estimated. The accumulation index of plasma concentration was calculated at steady state compared to the first dose.

## Results

Intravenous AS manufactured to be cGMP compliant (currently available through the CDC) was provided to 24 healthy volunteers aged 20–55 years (mean ± SD: 36.1 ± 12.4) and weighing 78.4 ± 13.2 kg who were enrolled in this study.

### Tolerability and safety

In this study, 24 healthy volunteers divided into three groups were treated with multiple intravenous infusions (a total of three doses) of AS with PBS buffer, and six volunteers were administered the same volume of vehicle. For each subject, adverse events (AEs) were recorded throughout the post-dosing period. Daily intravenous treatments with the WRAIR formulation of AS for three days were well tolerated in healthy volunteers at doses up to 8 mg/kg. There were no subject drop-outs for adverse events or other treatment related issues. A dose-related decrease in reticulocyte count was noted that peaked four days after dosing and returned to normal by study day 7 in most cases, and day 10 in all patients. There were no other clinically significant laboratory abnormalities detected. No deleterious haemodynamic or ECG effects were seen. A transient, reversible sensation of altered or unusual taste, which lasted less than 30 minutes in all cases, was associated with higher doses of AS. All remaining side effects were generally mild and all were reversible.

### Compartmental analysis of artesunate

**Figure 1:**
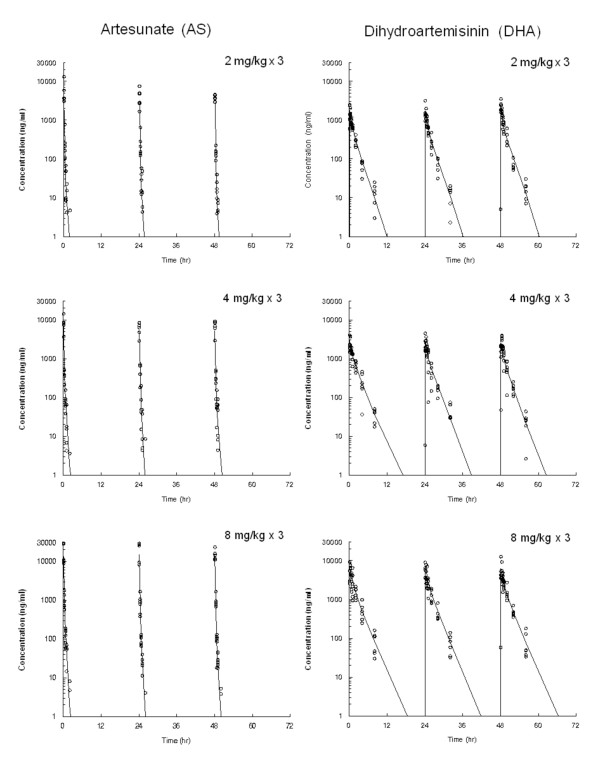
Mean plasma concentration-time profiles of artesunate (AS, left) and dihydroartemisinin (DHA, right), an active metabolite of AS, measured by LC-MS/MS (markers) and computer pharmacokinetic fitting curves (solid-line) following multiple intravenous dosage with 2 min short-term infusion of AS at 2 (top), 4 (middle), and 8 (bottom) mg/kg in healthy volunteers. (n = 6 for each dose cohort).

**Figure 2:**
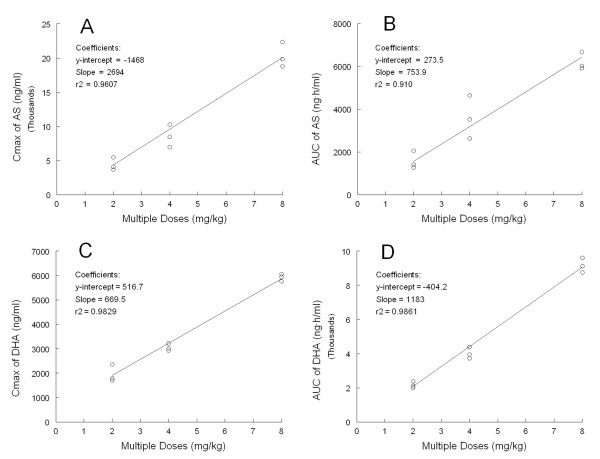
**Correlations (r**^**2**^ **= 0.910-0.986) are shown between multiple intravenous doses and peak concentration (C**_**max**_**) or area under the curve (AUC) with artesunate (AS) or dihydroartemisinin (DHA), an active metabolite from AS. A**: mean samples (markers) were taken from C_max_ of AS; **B**: mean values were taken from AUC of AS; **C**: mean samples were taken from C_max_ of DHA; and **D**: mean values were taken from AUC of DHA in all three dose groups (2, 4, and 8 mg/kg) after AS treatments daily for three days. The line represents linear regression whose statistical parameters are shown in the inset (A-D).

WinNonlin analysis resulted in a two-compartment model of intravenous infusion with first-order elimination, which best fitted the set of observations of intravenous AS in the present trials [20]. The plasma concentration and the final PK parameters estimates for the IV infusion in two-compartment model at daily dose of 2, 4, and 8 mg/kg for three days are shown in Figure 1 and Table 1, respectively. After the day 1 short term infusion (2 min), high plasma peak concentrations (C_max_) were reached, with mean C_max_ values ranging from 28411 ng/ml for the 2 mg/kg dose cohort to 63677 ng/ml for the 8 mg/kg dose cohort. The mean C_max_ and AUC_inf_ for AS in the subjects with the higher dose were roughly double when going from the 2 to the 4 mg/kg cohort and again when going from the 4 to the 8 mg/kg cohort and increased in proportion (r^2^ = 0.910-0.961) to the dose in all three dose groups (Figure 2, top).

**Table 1:** Mean (CV%) pharmacokinetic parameters of artesunate (AS) by intravenous-infusion compartment modeling (CA) following multiple intravenous administrations of 2, 4, and 8 mg/kg daily for 3 days with a short-term infusion (2 min) in healthy volunteers (n = 6 per group)*

**Date of dose**	**PK Parameters**	**2 mg/kg (n = 6)**	**4 mg/kg (n = 6)**	**8 mg/kg (n = 6)**
Day 1	C_max_ (ng/ml)	28411 (59)	40574 (68)	63677 (55)
	T_max_ (min)	2	2	2
	AUC_inf_ (ng·h/ml)	2051 (66)	4640 (69)	6022 (48)
	t_1/2 __elimination_ (h)	0.16 (52)	0.21 (46)	0.20 (40)
	CL_obs (ml/min/kg)	21.19 (33)	19.39 (48)	27.87 (51)
	Vss_obs (ml/kg)	103.60 (48)	104.34 (80)	187.99 (89)
	MRT (h)	0.08 (47)	0.08 (38)	0.10 (37)
Day 2	C_max_ (ng/ml)	17920 (57)	34387 (50)	74588 (45)
	T_max_ (min)	2	2	2
	AUC_inf_ (ng·h/ml)	1410 (49)	2628 (30)	6669 (47)
	t_1/2 __elimination_ (h)	0.19 (51)	0.23 (67)	0.19 (19)
	CL_obs (ml/min/kg)	29.50 (45)	27.26 (28)	26.66 (64)
	Vss_obs (ml/kg)	185.24 (57)	139.76 (51)	129.10 (68)
	MRT (h)	0.08 (35)	0.08 (29)	0.08 (8)
Day 3	C_max_ (ng/ml)	16487 (42)	51164 (61)	67613 (83)
	T_max_ (min)	2	2	2
	AUC_inf_ (ng·h/ml)	1270 (26)	3518 (51)	5916 (61)
	t_1/2 __elimination_ (h)	0.15 (40)	0.20 (46)	0.20 (60)
	CL_obs (ml/min/kg)	27.87 (26)	23.02 (35)	28.91 (44)
	Vss_obs (ml/kg)	122.00 (42)	122.17 (49)	164.58 (55)
	MRT (h)	0.07 (18)	0.08 (42)	0.09 (24)
	Accumulation Index (AUC_D3/D1_)	0.81 (56)	0.91 (45)	1.01 (31)
	P value (T-Test, AUC_D3:D1_)	0.104	0.289	0.459
	Total AUC_D1-D3_ (ng·h/ml)	4731 (40)	10787 (17)	18606 (45)

Data are presented as arithmetic mean (CV%); NCA = non-compartment analysis (WinNonlin 5.2 Version with non-compartment model 202, IV-infusion); CA = compartment analysis (WinNonlin 5.2 Version with compartment model 10, IV-infusion); MRT = mean residence time.

After the injection of 2 mg/kg of AS daily for three days, the mean AUC obtained with these doses ranged from 2,051 ng·h/ml on day 1 to 1,270 ng·h/ml on day 3 with a mild decrease, but no significant change statistically (p = 0.104), and the accumulation index was 0.81 (Table 1). Similar data were found in the groups of 4 and 8 mg/kg volunteers, and the accumulation indices were 0.80 for the 4 mg/kg subjects and 1.01 for the 8 mg/kg volunteers. No statistically significant variations (p > 0.289) of the accumulation or reduction of AS concentration were observed on day 3 in comparison to day 1 of treatment in plasma. The mean elimination half-lives ranged between 0.15 and 0.21 hr, and the mean residence time ranged from 0.07 and 0.08 hr during the three days of treatment following 2, 4, and 8 mg/kg doses injected (Table 1). Plasma total clearance (Cl) and volume of distribution during steady state (Vss) were all in a similar range without significant variation. Based on previous infusion PK analysis [20], the PK results indicated that the parameters of AS can be calculated only with compartment modelling and the data set is model-dependent.

### Non-compartmental analysis of dihydroartemisinin

The plasma concentrations of DHA, an active metabolite of AS, after short-term infusion at multiple doses of 2, 4, and 8 mg/kg are shown in Figure 1. Table 2 shows the mean parameters (CV%) in a first approach for the non-compartmental analysis (NCA). The mean C_max_ and AUC_inf_ for DHA were increased in a dose-dependent manner in all three dose cohorts. Mean peak concentrations of DHA over the three days ranged from 1735–2358 ng/ml in the 2 mg/kg cohort, 2923–3018 ng/ml in the 4 mg/kg cohort, and 5762–6056 ng/ml in the 8 mg/kg cohort. Mean AUC_inf_ values over three days ranged from 2021–2385 ng·h/ml in the 2 mg/kg cohort, 3740–4391 ng·h/ml in the 4 mg/kg cohort, and 8732–9697 ng·h/ml in the 8 mg/kg cohort (Table 2). In no cohort were statistically significant C_max_ and AUC differences observed between Day 1 and Day 3 (Table 2). Mean T_max_ was revealed to fall in a range of 0.16-0.29 hr and the elimination t_1/2_ s was in a range of 1.19-1.91 hr. The mean body clearance over bioavailability (CL/F) and volume of distribution at steady state over bioavailability (Vss/F) ranged from 14.63-19.37 ml/min/kg and 1,691-2,377 ml/kg, respectively. A dose-dependent increase of C_max_ and AUC_inf_ for DHA in the subjects were shown to correlate well (r^2^ = 0.983-0.986) to the dose in all three dose cohorts (Figure 2).

**Table 2:** Mean (CV%) PK parameters of dihydroartemisinin (DHA), an active metabolite of artesunate (AS), by non-compartment analysis (NCA) following multiple intravenous administrations of AS at dose 2, 4, and 8 mg/kg daily for 3 days with a short-term infusion (2 min) in healthy volunteers* (n = 6 per group)

**Date of dose**	**PK Parameters**	**2 mg/kg (n = 6)**	**4 mg/kg (n = 6)**	**8 mg/kg (n = 6)**
Day 1	C_max_ (ng/ml)	1735 (31)	3015 (29)	6056 (40)
	T_max_ (hr)	0.29 (45)	0.25 (55)	0.25 (59)
	AUC_inf_ (ng·h/ml)	2121 (18)	4391 (22)	9697 (37)
	t_1/2 __elimination_ (h)	1.19 (16)	1.74 (59)	1.42 (21)
	CL/F (ml/min/kg)	16.18 (19)	15.92 (26)	15.51 (37)
	Vss/F (ml/kg)	1634 (14)	2377 (62)	1870 (39)
	MRT (h)	1.41 (12)	1.91 (22)	1.81 (16)
Day 2	C_max_ (ng/ml)	1710 (41)	2923 (35)	5943 (35)
	T_max_ (hr)	0.20 (63)	0.25 (55)	0.22 (69)
	AUC_inf_ (ng·h/ml)	2012 (27)	3740 (28)	8732 (35)
	t_1/2 __elimination_ (h)	1.59 (55)	1.42 (22)	1.91 (60)
	CL/F (ml/min/kg)	17.55 (24)	19.37 (35)	16.96 (35)
	Vss/F (ml/kg)	2224 (31)	2249 (21)	2525 (35)
	MRT (h)	1.53 (24)	1.65 (20)	2.09 (36)
Day 3	C_max_ (ng/ml)	2358 (26)	3018 (25)	5762 (59)
	T_max_ (hr)	0.16 (63)	0.29 (45)	0.29 (45)
	AUC_inf_ (ng·h/ml)	2385 (24)	3960 (19)	9205 (33)
	t_1/2 __elimination_ (h)	1.31 (16)	1.24 (20)	1.34 (12)
	CL/F (ml/min/kg)	14.63 (22)	17.49 (23)	15.60 (27)
	Vss/F (ml/kg)	1691 (33)	1895 (34)	1776 (25)
	MRT (h)	1.39 (6)	1.57 (16)	1.78 (11)
	Accumulation Index(AUC_D3/D1_)	1.14 (21)	0.93 (24)	0.98 (15)
	P value (T-Test, AUC_D3:D1_)	0.140	0.157	0.233
	Total AUC_D1-D3_	6520 (18)	12092 (20)	27635 (34)
	Ratio of AUC_DHA_/AUC_AS_	1.46 (23)	1.15 (28)	1.65 (39)
	Ratio of C_max AS_/C_max DHA_	3.08 (45)	3.71 (47)	3.78 (49)

Data are presented as arithmetic mean (CV%); NCA = non-compartment analysis (WinNonlin 5.2 Version with non-compartment model 202, IV-infusion); CA = compartment analysis (WinNonlin 5.2 Version with compartment model 10, IV-infusion); MRT = mean residence time.

### Pharmacokinetic comparison of artesunate and dihydroartemisinin parameters

The conversion data of AS to DHA is presented in Table 2. In all three dose cohorts, the plasma concentration (AUC) of DHA was more than that of AS with a range of 1.15-1.65 ratio of AUC_DHA/AS_, but the peak concentration (C_max_) of AS was much higher than that of DHA with a range of 3.08-3.78 ratio of C_max__AS/DHA_. DHA also revealed a longer half-life of 1.19-1.91 hr, which was 7.8 – 8.2 fold longer than shorter half-lives of AS with 0.15 – 0.21 hr. If the AUC values of AS and DHA were combined, the total combined AUC calculated was increased proportionally to the AS climbing doses as well in all three dose cohorts (Table 2).

## Discussion

In this study, the PK and possible side effects of an intravenous infusion of AS were investigated in healthy volunteers. Up to the highest dose of 8 mg/kg daily for three days, intravenous AS was well tolerated and no serious side effects were observed. A dose-related decrease in reticulocyte count was noted that peaked four days after dosing and returned to normal by study day 7 in most cases. There were no other clinically significant laboratory abnormalities detected. No deleterious haemodynamic or ECG effects were seen.

Two methods to determine PK parameters are the compartmental (model-dependent) and non-compartment (model-independent) PK analyses, which are perfectly acceptable by the US FDA for new drug application [21]. In a previous clinical trial [20], PK evaluation with both non-compartmental analysis (NCA) and compartment analysis (CA) were performed for both AS and its active metabolite, DHA. The results showed that for all PK parameters of DHA there were no significant differences in C_max_, AUC, and other parameters between the NCA and CA analyses, indicating that the PK modelling for DHA is model-independent. This suggests that either modelling method can be used for PK analysis. However, it is not suitable for the PK evaluation of AS with a short-term intravenous infusion. When using NCA for the parent drug, the AUC calculation of AS will lose the contribution of the C_max_ at the end of the infusion at two minutes because the C_max_ was only estimated by a compartmental model of IV-infusion at the ending time of infusion. In the previous report, the AUC was under calculated by 28-40% using the NCA method when compared to CA analysis [20]. Therefore, only CA analysis can fulfill the PK evaluation of AS with the infusion administration. After comparison to the NCA modelling method, the two-compartment, IV-infusion model was appropriately described by the disposition PK data of intravenous AS, which should be fitted in the present study by a model-dependent algorithm.

Similar to the literature, the results showed that AS is rapidly converted to DHA, which was detected in all volunteers' plasma until six hours, while the parent drug was undetectable at timepoints as early as one to two hr after each short-term infusion. *In vitro*, DHA is similar to AS [1], and three to five-fold more active and more toxic than the other artemisinin derivatives [1,2]. Although the plasma concentration of DHA was superior to that of AS with a range of 1.15 to 1.65 ratio of AUC_DHA/AS_, the peak concentration of AS was higher than that of DHA with 3.08 to 3.78 ratio of C_max__AS/_ C_max DHA_ (Table 2). It is known that DHA has a longer half-life of 1.23 to 1.63 hr, which is 7.8 to 8.2-fold longer than AS with a short half-life of 0.15 to 0.21 hr. Therefore, the effectiveness of AS has been attributed to itself and to its rapid and extensive hydrolysis to DHA [5-8].

Mean AUC of AS obtained with these doses ranged on day 3 from 1,270-5,916 ng·h/ml, which was less than that observed on day 1 (2,051-6,022 ng·h/ml), but no significant change was shown in these data statistically. Likewise for DHA, no significant change of AUCs was found between day 3 (2,385-9,205 ng·h/ml) and day 1 (2,121-9,697 ng·h/ml) following the multiple intravenous injections. These results indicate that no significant plasma concentration was found in these healthy volunteers after the administration of three intravenous doses due to only three days dosing with low power in the present study. However, after oral dosing, four artemisinin drugs (artemisinin, artemether, AS, and DHA) have revealed declining concentrations in plasma during multiple treatments in malaria patients and healthy subjects. The C_max_ and AUC values for AS were markedly reduced, about one-third to one-seventh on the last dose day as compared to the first day with minimal 5 days dosing [22-26]. The decrease in plasma concentration time during multiple treatments is indicative of an increase in metabolic capacity due to auto-induction of hepatic drug-metabolizing enzymes in patients [22,23] and in healthy subjects [24-26].

In the present report, the peak concentration (C_max_) of AS was much higher than that of DHA with a range of 3.08–3.78 ratio of C_max__AS/DHA_. The high peak concentrations with these doses (16,487-74,588 ng/ml) of AS provide a great advantage to rapidly kill malaria parasites. PK/PD evaluations demonstrate that the rapid efficacy of artemisinins is principally due to the drug peak concentration (C_max_) because only correlative with parasite killing is the peak concentration among the five artemisinins, indicating AS is superior in terms of both PK and PD following either oral or intravenous administration. Other pharmacokinetic parameters, such as drug exposure level (AUC) and drug exposure time (half-life) were not correlated to the parasite killing and tend to be of minor importance [15,16]. More clinical observations have been reported demonstrating the fast pharmacodynamic properties of these artemisinins have been relatively addressed with a PK/PD evaluation [27,28].

In the previous clinical trials, the mean peak level (C_max_) of intravenous AS was achieved at 11,343 ng/ml and reduced the duration of the lag phase of the parasitaemia curve to 1.92 hr, which was vastly decreased when compared with oral AS with C_max_ of 1052 ng/ml (2.81 hr lag time) and the other four artemisinins (4.03-8.89 hr lag time) with C_max_ range of 74.9 – 588.0 ng/ml [15]. The summary indicated that the parasite-killing effect of AS injection was very fast and efficient showing a mean time of 3.18 hr for clearance of half the parasitaemia (PC_50_), the lowest PC_50_ when compared to oral AS and other artemisinins, which demonstrated a PC_50_ of 8.48-19.68 hr. Oral AS and the other four artemisinins (oral DHA, oral artemisinin, intramuscular artemether and arteether) showed a slower killing efficacy because their administration resulted in a lower peak concentration ranging from 74.9-1,052 ng/ml [15,16].

These facts together indicate that AS is a superior anti-malarial agent in terms of PK/PD performance. Intravenous AS can provide the highest peak concentration and a very short exposure time, while oral AS can also provide higher peak level and short exposure times; the higher peak level of injectable AS plays a role in parasite elimination rapidly and the short exposure time allows patients to avoid fatal neurotoxicity [29], and also prevents the rise of parasite resistance [30]. All of the evidence shows that higher plasma concentration (C_max_ and AUC), especially the peak concentration (C_max_), will greatly increase the efficacy and clinical therapeutic potential of artemisinin drugs. As discussed above, from the severity of these complications, *P. falciparum* malaria constitutes a medical emergency, and appropriate treatment should be initiated as soon as infection is suspected. This study suggests that intravenous high dose AS therapy is most appropriate treatment for patients with severe malaria. It is known that 84% of all malaria-related deaths occur within 24 hours of hospital admission in African children [31]. Therefore, first exposure concentrations of artemisinin drugs are clinically very important.

In conclusion, the study presented here underlines the need for appropriate PK analysis of AS and its metabolite, DHA. Due to a short-term duration of intravenous infusion, PK analyses of the data observed after intravenous AS administration is more suitably fitted to model-dependent rather than model-independent algorithms. Injectable AS is a superior anti-malarial agent in terms of PK performance with the highest peak concentration and the shortest exposure time. The higher peak level of AS concentration plays a crucial role in eliminating parasites rapidly and the short exposure time allows patients to avoid fatal neurotoxicity. The present data show that the administration of injectable AS at a high dose of 8 mg/kg is safe, and the use of higher doses may increase the probability of success when used in patients with larger variability in PK and PD [16,32].

## Abbreviations

AS: Artesunate; DHA: Dihydroartemisinin; PK: Pharmacokinetics; cGMP: Current Good Manufacturing Practices.

## Competing interests

The authors are all US Government employees and there is no copyright to transfer and they declare no conflict of interest.

## Authors’ contributions

RSM, LC and PW designed the studies and interpreted safety data, KL, GS and BS performed the clinical trial, VM performed LC-MS/MS analysis, QL analysed the PK data and prepared the manuscript. All authors read and approved the final manuscript.

## References

[B1] Li Q Gerena L Xie L Zhang J Kyle D Milhous W Development and validation of flow cytometric measurement for parasitemia in cultures of P. falciparum vitally stained with YOYO-1 Cytometry A 2007 71 297 307 17279569 10.1002/cyto.a.20380

[B2] Li Q Mog SR Si YZ Kyle DE Gettayacamin M Milhous WK Neurotoxicity and efficacy of arteether related to its exposure times and exposure levels in rodents Am J Trop Med Hyg 2002 66 516 525 12201585 10.4269/ajtmh.2002.66.516

[B3] McLean WG Ward SA In vitro neurotoxicity of artemisinin derivatives Med Trop (Mars) 1998 58 3 Suppl 28 31 10212894

[B4] Skinner TS Manning LS Johnston WA Davis TM In vitro stage-specific sensitivity of Plasmodium falciparum to quinine and artemisinin drugs Int J Parasit 1996 26 519 525 10.1016/0020-7519(96)89380-5 8818732

[B5] Byakika-Kibwika P Lamorde M Mayito J Nabukeera L Mayanja-Kizza H Katabira E Hanpithakpong W Obua C Pakker N Lindegardh N Tarning J de Vries PJ Merry C Pharmacokinetics and pharmacodynamics of intravenous artesunate during severe malaria treatment in Ugandan adults Malar J 2012 11 132 10.1186/1475-2875-11-132 22540954 PMC3489518

[B6] Davis TM Phuong HL Ilett KF Hung NC Batty KT Phuong VD Powell SM Thien HV Binh TQ Pharmacokinetics and pharmacodynamics of intravenous artesunate in severe falciparum malaria Antimicrob Agents Chemother 2001 45 181 186 10.1128/AAC.45.1.181-186.2001 11120963 PMC90258

[B7] Nadjm B Behrens RH Malaria: an update for physicians Infect Dis Clin North Am 2012 26 243 259 10.1016/j.idc.2012.03.010 22632637

[B8] Navaratnam V Mansor SM Sit NW Grace J Li QG Olliaro P Pharmacokinetics of artemisinin-type compounds Clin Pharmacokinet 2000 39 255 270 10.2165/00003088-200039040-00002 11069212

[B9] Janssens B van Herp M Goubert L Chan S Uong S Nong S Socheat D Brockman A Ashley EA Van Damme W A randomized open study to assess the efficacy and tolerability of dihydroartemisinin-piperaquine for the treatment of uncomplicated falciparum malaria in Cambodia Trop Med Int Health 2007 12 251 259 10.1111/j.1365-3156.2006.01786.x 17300633

[B10] Malik EM Mohamed TA Elmardi KA Mowien RM Elhassan AH Elamin SB Mannan AA Ahmed ES From chloroquine to artemisinin-based combination therapy: the Sudanese experience Malar J 2006 5 65 10.1186/1475-2875-5-65 16879742 PMC1590042

[B11] Rosenthal PJ Artesunate for the treatment of severe falciparum malaria N Engl J Med 2008 358 1829 1836 10.1056/NEJMct0709050 18434652

[B12] Kreeftmeijer-Vegter AR van Genderen PJ Visser LG Bierman WF Clerinx J van Veldhuizen CK de Vries PJ Treatment outcome of intravenous artesunate in patients with severe malaria in the Netherlands and Belgium Malar J 2012 11 102 10.1186/1475-2875-11-102 22462806 PMC3364861

[B13] Dondorp A Nosten F Stepniewska K Day N White N South East Asian Quinine Artesunate Malaria Trial (SEAQUAMAT) group. Artesunate versus quinine for treatment of severe falciparum malaria: a randomised trial Lancet 2005 366 717 725 16125588 10.1016/S0140-6736(05)67176-0

[B14] Dondorp AM Fanello CI Hendriksen IC Gomes E Seni A Chhaganlal KD Bojang K Olaosebikan R Anunobi N Maitland K Kivaya E Agbenyega T Nguah SB Evans J Gesase S Kahabuka C Mtove G Nadjm B Deen J Mwanga-Amumpaire J Nansumba M Karema C Umulisa N Uwimana A Mokuolu OA Adedoyin OT Johnson WB Tshefu AK Onyamboko MA Sakulthaew T Artesunate versus quinine in the treatment of severe falciparum malaria in African children (AQUAMAT): an open-label, randomised trial Lancet 2010 376 1647 1657 10.1016/S0140-6736(10)61924-1 21062666 PMC3033534

[B15] Li Q Weina P Milhous WK Pharmacokinetic and pharmacodynamic profiles of rapid-acting artemisinins in the antimalarial therapy Current Drug Therapy 2007 2 210 223 10.2174/157488507781695649

[B16] Li Q Weina PJ Li Q, Weina PJ Antimalarial drugs: age of the artemisinins 2010 New York: Nova Science Publishers Inc 243 328

[B17] Nosten F Ashley E McGready R Price R We still need artesunate monotherapy BMJ 2006 333 45 16809721 10.1136/bmj.333.7557.45 PMC1488766

[B18] Jones KL Donegan S Lalloo DG Treating severe malaria: artesunate or quinine? Indian Pediatr 2008 45 41 42 18250504

[B19] Hess KM Goad JA Arguin PM Intravenous artesunate for the treatment of severe malaria Ann Pharmacother 2010 44 1250 1258 10.1345/aph.1M732 20551300

[B20] Li Q Cantilena LR Leary KJ Saviolakis GA Miller RS Melendez V Weina PJ Pharmacokinetic profiles of artesunate after single intravenous doses at 0.5, 1, 2, 4, and 8 mg/kg in healthy volunteers: a phase I study Am J Trop Med Hyg 2009 81 615 621 10.4269/ajtmh.2009.09-0150 19815876

[B21] FDA Guidance for Industry General considerations for pediatric pharmacokinetic studies for drugs and biological products 1998 http://www.fda.gov/cder/guidance/1970dft.pdf

[B22] Ashton M Sy ND Gordi T Hai TN Thach DC Huong NV Johansson M Coeng LD Evidence for time-dependence artemisinin kinetics in adults with uncomplicated malaria Pharm Pharmacol Lett 1996 6 127 130

[B23] Khanh NX de Vries PJ Ha LD van Boxtel CJ Koopmans R Kager PA Declining concentrations of dihydroartemisinin in plasma during 5-day oral treatment with artesunate for falciparum malaria Antimicrob Agent Chemother 1999 43 690 692 10.1128/aac.43.3.690 PMC89184 10049291

[B24] van Agtmael MA Shan CQ Jiao XQ Mull R van Boxtel CJ Multiple dose pharmacokinetics of artemether in Chinese patients treated for falciparum malaria Int J Antimicrob Agents 1999 12 151 158 10.1016/S0924-8579(99)00063-1 10418761

[B25] Ashton M Hai TN Sy ND Artemisinin pharmacokinetics is time-dependent during repeated oral administration in healthy male adults Drug Metab Dispos 1998 26 25 27 9443848

[B26] Park BK O’Neill PN Maggs JL Pirmohamed M Safety assessment of peroxide antimalarials: clinical and chemical perspectives Br J Clin Pharmacol 1998 46 521 529 9862239 10.1046/j.1365-2125.1998.00838.x PMC1873802

[B27] Li Q Lugt CB Looareesuwan S Krudsood S Wilairatana P Vannaphan S Chalearmrult K Milhous WK Pharmacokinetic investigation on the therapeutic potential of artemotil (beta-arteether) in Thai patients with severe Plasmodium falciparum malaria Am J Trop Med Hyg 2004 71 723 731 15642961

[B28] Kyle DE Teja-Isavadharm P Li Q Leo K Pharmacokinetics and pharmacodynamics of qinghaosu derivatives: how do they impact on the choice of drug and the dosage regimens? Med Trop (Mars) 1988 58 3 Suppl 38 44 10212896

[B29] Li Q Hickman MR Toxicokinetic and toxicodynamic (TK/TD) evaluation to determine and predict the neurotoxicity of artemisinins Toxicology 2011 279 1 9 10.1016/j.tox.2010.09.005 20863871

[B30] Dondorp AM Yeung S White L Nguon C Day NP Socheat D von Seidlein L Artemisinin resistance: current status and scenarios for containment Nat Rev Microbiol 2010 8 272 280 20208550 10.1038/nrmicro2331

[B31] Marsh K Forster D Waruiru C Mwangi I Winstanley M Marsh V Newton C Winstanley P Warn P Peshu N Indicators of life-threatening malaria in African children N Engl J Med 1995 332 1399 1404 10.1056/NEJM199505253322102 7723795

[B32] Day N Dondorp AM The management of patients with severe malaria Am J Trop Med Hyg 2007 77 6 Suppl 29 35 18165472

